# Novel characterization of endogenous transient receptor potential melastatin 3 ion channels from Gulf War Illness participants

**DOI:** 10.1371/journal.pone.0305704

**Published:** 2024-06-25

**Authors:** Sonya Marshall-Gradisnik, Etianne Martini Sasso, Natalie Eaton-Fitch, Peter Smith, James N. Baraniuk, Katsuhiko Muraki

**Affiliations:** 1 The National Centre for Neuroimmunology and Emerging Diseases, Griffith University, Gold Coast, QLD, Australia; 2 Consortium Health International for Myalgic Encephalomyelitis, National Centre for Neuroimmunology and Emerging Diseases, Griffith University, Gold Coast, QLD, Australia; 3 School of Pharmacy and Medical Sciences, Griffith University, Gold Coast, QLD, Australia; 4 Clinical Medicine, Griffith University, Gold Coast, QLD, Australia; 5 Department of Medicine, Georgetown University, Washington, DC, United States of America; 6 Laboratory of Cellular Pharmacology, School of Pharmacy, Aichi-Gakuin University, Nagoya, Japan; Weizmann Institute of Science, ISRAEL

## Abstract

Gulf War Illness (GWI) is a chronic condition characterized by multisystem symptoms that still affect up to one-third of veterans who engaged in combat in the Gulf War three decades ago. The aetiology of GWI is mainly explained by exposure to multiple toxic agents, vaccines, and medications. As there is a significant overlap in symptoms between GWI and Myalgic Encephalomyelitis/Chronic Fatigue Syndrome (ME/CFS), the objective of this study was to investigate a biomarker widely reported in Natural Killer (NK) cells from ME/CFS patients, the Transient Receptor Potential Melastatin 3 (TRPM3) ion channel. NK cells from 6 healthy controls (HC) and 6 GWI participants were isolated, and TRPM3 function was assessed through whole-cell patch-clamp. As demonstrated by prior studies, NK cells from HC expressed typical TRPM3 function after pharmacomodulation. In contrast, this pilot investigation demonstrates a dysfunctional TRPM3 in NK cells from GWI participants through application of a TRPM3 agonist and confirmed by a TRPM3 antagonist. There was a significant reduction in TRPM3 function from GWI than results measured in HC. This study provides an unprecedented research field to investigate the involvement of TRP ion channels in the pathomechanism and potential medical interventions to improve GWI quality of life.

## Introduction

Gulf War Illness (GWI) is a chronic, debilitating, and multisystem disorder that affects people who served in the Gulf War (GW). In 1990 and 1991, almost one million defence personnel from 41 countries engaged in combat in the GW, where they were exposed to diverse known and unknown/undefined hazardous environmental stressors, biological and chemical agents (insecticide, pesticide, insect repellent, organophosphate nerve agents, smoke from oil-well fires, depleted uranium) [1–4]. Prophylactic strategies, several mandatory vaccines and drugs were also administered concomitantly to reduce conflict risk, such as botulinum toxoid and anthrax vaccines and pyridostigmine bromide to protect from nerve agent exposures [5–8] in addition to the hypervigilance and mental stress that life-threatening military conflict brings.

Despite the fact that defence personnel returned from the GW more than three decades ago, it is estimated that from 25% to 32% of GW veterans continue to suffer from health issues and disabling symptoms likely caused by exposures during the GW [4,9,10]. In general, GWI symptomatology includes chronic fatigue, pain, inflammation, sleep disturbances, neurological and cognitive impairment, gastrointestinal and respiratory disorders, and post-exertional malaise, but not all affected individuals develop the entire array of symptoms, which makes diagnosis difficult [9,11–13]. Currently, the most recommended consensus definitions for GWI are: Chronic multisymptom illness from the Centers for Disease Control and Prevention (CDC) [14] and Kansas definition [15].

To date, GWI aetiology remains not completely understood, however, scientific evidence suggests the most reasonable cause of GWI is the exposure to the abovementioned combination of toxicant agents (insecticide, pesticide, insect repellent, organophosphate nerve agents, smoke from oil-well fires, depleted uranium) and prophylactic strategy [9,16,17]. Interestingly, GWI symptoms spectrum significantly overlaps Myalgic Encephalomyelitis/Chronic Fatigue Syndrome (ME/CFS), fibromyalgia and long COVID-19, all conditions in which complex pathomechanisms are only partially defined, including some GWI patients who meet the clinical criteria for ME/CFS and Fibromyalgia [6,13,18,19]. A remarkable feature of ME/CFS is the post-exertional neuroimmune exhaustion caused by an inappropriate response to stressors and involves symptoms exacerbated following mild physical or mental activity [20–22], a characteristic also reported in GWI patients [12,23].

Our previous studies have demonstrated the role of Transient Receptor Potential (TRP) ion channels in the pathophysiology of ME/CFS which also promoted research into novel therapies [24–29]. The Mammalian TRP ion channels family is compounded by six subfamilies totaling 28 members: ankyrin (TRPA), canonical (TRPC), melastatin (TRPM), mucolipin (TRPML), polycystin (TRPP), and vanilloid (TRPV) [30–32]. Furthermore, a large number of the TRP superfamily members are highly sensitive to diverse chemical, physical and biological stimuli, therefore these ion channels act as molecular sensors to perceive modifications in the internal and external environment for the purpose of maintaining homeostasis [33–38]. In addition, many TRP channels participate in the regulation of calcium (Ca^2+^) signalling to preserve cellular homeostasis. Overall, TRP channels also contribute to crucial processes, including neurogenesis, plasticity, immune functions, inflammation control, cell proliferation and survival, and pain perception [32,37,39–45].

Although GWI symptoms are the result of the long-term effects of exposure during military service, and the potential for TRP channels to be modulated by various environmental and toxic stimuli, to our knowledge this is the first study to analyse a TRP channel function in veterans with GWI. We specifically selected TRPM3 as the first TRP channel to be investigated in the GWI pathomechanism due to TRPM3’s association with ME/CFS, even though other TRP channels may also be associated with the symptomatology presented by patients with GWI. Hence, in this pilot study, the aim was to evaluate TRPM3 ion channel activity in NK cells from GWI participants in comparison with the same cells from healthy controls (HC).

## Materials and methods

### Participant characteristics

Participants diagnosed with GWI and HC participants were recruited between 06^th^ June and 30^th^ November 2023 to be included in this study. The GWI group was composed of six Australian males who engaged in combat at the GW, were diagnosed with GWI previously, and met CDC Case Definition [14] and Kansas criteria [15] for GWI. Six HC male participants were selected for this study, all those reported to be in good health, absence of illness and no fatigue episodes. All volunteers were between 18 and 65 years and non-smokers. Participants were excluded from this investigation if they were previously diagnosed with a chronic illness or reported alcohol abuse, use of opioids, medication, or supplements that interfere with TRPM3 ion channels or Ca^2+^ signalling. However, participants had the option to cease taking conflicting medications in accordance with the half-life of pharmacological compounds and if authorised by their physician. This investigation was approved by the Griffith University Human Research Ethics Committee (GU HREC 2022/666) and all participants provided their written consent.

### Participant symptoms and disability

All participants completed a questionnaire created by the National Centre for Neuroimmunology and Emerging Diseases (NCNED) to provide their medical history, sociodemographic background, illness symptoms, and disability information. To assess disability data the questionnaire includes the World Health Organization Disability Assessment Schedule (WHODAS).

In this study, symptoms from people with GWI were classified in ten subtypes: (1) cognitive difficulties (e.g. cognitive overload, confusion, disorientation, impaired concentration, forgetfulness and memory problems); (2) pain (e.g. headaches, muscle aches and multi-joint pain); (3) sleep disturbances (e.g. unrefreshing sleep, frequent awakenings, prolonged sleep, reversed sleep cycle); (4) cardiovascular symptoms (e.g. orthostatic intolerance, cardiac arrhythmias, heart palpitations, light headedness and dizziness); (5) respiratory symptoms (e.g. air hunger, difficulty breathing); (6) thermostatic intolerances (e.g. subnormal body temperature, abnormal sweating episodes, hot flushes and cold extremities); (7) neurosensory or perceptual symptoms (e.g. inability to focus vision, impaired depth perception, sensitivity to touch, light, odour, taste, sound, vibration and poor balance or coordination); (8) urinary changes (e.g. changes to urination frequency and urgency to urinate); (9) immune disturbances (e.g. sore throat, tender lymph nodes, new allergies/sensitivities); and (10) gastrointestinal disturbances (e.g. nausea, abdominal pain, bloating, diarrhoea and irritable bowel syndrome).

The WHODAS indicates the level of disability from each participant and data is combined by groups. WHODAS is subdivided into seven domains of life: (1) Communication and understanding; (2) Mobility; (3) Self-care; (4) Interpersonal connections; (5) Life activities; (6) Work or School participation; and (7) Participation in society. WHODAS items were scored on a five-point scale (none, mild, moderate, severe, and extreme or cannot do). The subscale scores were determined in accordance with the WHODAS 2.0 manual, first converting each item score into the corresponding, predefined weighted values [46]. Scores converted from 0% to 100%, disability are inversely proportional to the scale, whereby lower scores indicate less disability and correspond 100% to full disability [47].

### Peripheral blood mononuclear cell and natural killer cell isolation

Each participant donated between 40 ml and 84 ml of whole blood. All blood collections were conducted by a qualified phlebotomist, via venepuncture, using ethylenediaminetetraacetic acid (EDTA) tubes. A sample of 4 ml of whole blood from each participant was sent to a pathology laboratory for full blood count (FBC).

The remaining whole blood samples were used to isolate peripheral blood mononuclear cells (PBMCs) by centrifugation over a density gradient medium (Ficoll-Paque Premium, GE Healthcare, Uppsala, Sweden). PBMCs total cell count, live cell count and viability were assessed using trypan blue dye (Invitrogen, Carlsband, CA, USA) and automatic cell counter (TC20 Automated cell counter, Bio-Rad, Laboratories, Hercules, CA).

For NK cell isolations, PBMCs were adjusted for a concentration of 5×10^7^ cells/ml. NK cell isolations were conducted by immunomagnetic selection using an EasySep Negative Human NK Cell Isolation Kit (Stem Cell Technologies, Vancouver, BC, Canada).

Flow cytometry was performed to identify the NK cell purification from each NK cell isolation. Immediately after NK cell isolation, cells were incubated with CD56 APC (0.25g/20l) and CD3 PE Cy7 (0.25g/5l) monoclonal antibodies (Becton Dickinson (BD) Bioscience, San Jose, CA, USA) for 20 minutes in the dark at room temperature. NK cells were washed and resuspended in 350 ml of stain buffer (BD Bioscience, New Jersey, USA) and acquired at 10,000 events using the BD LSR- FortessaTM X-20 flow cytometer (BD Biosciences, San Diego, CA, USA). The NK cell population was then identified using phenotypic surface expression as CD3^-^CD56^+^. For this study, acceptable NK cells purity was ≥ 90%. S1 Fig shows and compares purity results from HC and GWI groups. Importantly, there was no statistical difference between groups.

### Electrophysiological experiments

The gold standard patch-clamp technique was conducted to determine TRPM3 ion channel activity in NK cells freshly isolated from HC and people with GWI. In this study, borosilicate glass capillaries (Harvard Apparatus, Holliston, MA, USA, GC150F-15, outside diameter = 1.5 mm, inside diameter = 0.86 mm) were pulled to obtain glass pipette (Sutter Instrumental, model P-97) and polished posteriorly (Narishige, Micro Forge MF-900). When filled with pipette solution, membrane resistance was 8 to 12 MΩ. A CV203BU head-stage (Molecular Devices, Sunnyvale, CA, USA) connected to a 3-way coarse manipulator and a micromanipulator (Narishige, Tokyo, Japan) were used in these experiments. To amplify and record electrical signals, an Axopatch 200B amplifier and pClamp 10.7 software (Molecular Devices, Sunnyvale, CA, USA) were used, with data filtered at 5 kHz and sampled digitally at 10 kHz via a Digidata 1440A analogue to digital converter (Molecular Devices, Sunnyvale, CA, USA). The voltage-ramp protocol was a step from a holding potential of +10 mV to -90 mV, followed by a 0.1 s ramp to +110 mV, before returning to +10 mV (repeated every 10 seconds). The liquid junction potential between the pipette and bath solutions (10 mV) was corrected and no leak current component was subtracted.

The intracellular pipette solution contained: 30 mM CsCl, 2 mM MgCl_2_, 110 mM L-Aspartic acid, 1 mM EGTA, 10 mM HEPES, 4 mM ATP disodium hydrate, 0.1 mM GTP sodium salt hydrate (pH = 7.2, adjusted with CsOH; Osmolality = 290 mOsm/L, adjusted with D-mannitol), filtered with 0.22 m membrane filter (Sigma-Aldrich, St. Louise, MO, USA), aliquoted and stored at -20°C. The possibility of chloride current involvement in TRPM3 assessment was minimized by using L-Aspartic acid in the intracellular pipette solution. The extracellular solution contained: 130 mM NaCl, 10 mM CsCl, 1 mM MgCl_2_, 1.5 mM CaCl_2_ 2H_2_O, 10 mM HEPES, (pH = 7.4, adjusted with NaOH; Osmolarity = 300 mOsm/L, adjusted with D-glucose) freshly prepared.

As previously validated by NCNED, pharmacological agents were included in the extracellular solution to assess TRPM3 ionic currents [26]. Briefly, a gravity perfusion system was used to apply extracellular solution for 50 seconds to establish a baseline current. Subsequently, extracellular solution containing 100 μM of PregS was added to stimulate TRPM3 ion channels for 2.5 minutes. Following on from this addition extracellular solution with 10 μM Ononetin and 100 μM of PregS was applied for another 2.5 minutes to block TRPM3 ion channels. After the conclusion of the drugs application, another cycle of 100 seconds of only extracellular solution was applied to remove the drugs. ATP and GTP were purchased from Sapphire Bioscience Reagents, PregS and Ononetin were purchased from Tocris Bioscience, while all other reagents and chemicals were ordered from Sigma-Aldrich. PregS and Ononetin were resuspended and stored in accordance with the manufacturer’s instructions. Electrophysiological experiments were conducted at room temperature (22–24°C).

All recordings were analysed individually by a blinded researcher and posteriorly data was reviewed one by one by another blinded researcher, as detailed in S2 Fig. Additionally, any unstable currents or chloride contamination was excluded from the analysis.

### Statistical analysis

Questionnaire data were analysed through the Statistical Package for the Social Sciences (SPSS) software, version 27 (IBM Corp, Armonk, NY, USA) and purity results with GraphPad Prism v9 (GraphPad Software Inc., La Jolla, CA, USA). For electrophysiological data analysis and data presentation, pCLAMP 10.7 software (Molecular Devices, Sunnyvale, CA, USA), Origin 2021 (OriginLab Corporation, Northampton, MA, USA), and GraphPad Prism version 9 were used. Shapiro-Wilk normality test was performed to identify the distribution of data. ROUT method was conducted to determine outliers and they were removed from analysis. The independent nonparametric Mann-Whitney U test was performed to identify the statistical significance between GWI and HC groups in PregS and Ononetin amplitude. The Fisher’s exact test (applying Bonferroni method) was conducted to determine statistical significance regarding sensitivity to Ononetin in NK cells. Significance was set at p < 0.05 and the data are presented as mean ± standard error of the mean (SEM) unless otherwise stated.

## Results

### Participant characteristics and full blood count

In general, there were no significant differences between GWI and HC participants regarding age (HC = 47.33 ± 9.24 and GWI = 52.33 ± 2.07), body mass index (BMI) (HC = 29.22 ± 4.20 and GWI = 25.93 ± 1.90), employment status and education level. An overview of participants’ features is detailed in Table 1.

**Table 1:** Participant characteristics.

		HC	GWI	P-value
Age (years)		47.33 ± 9.24	52.33 ± 2.07	0.180
BMI (kg/m2)		29.22 ± 4.20	25.93 ± 1.90	0.200
Employment Status	Full Time	5 (83.33%)	4 (66.66%)	0.400
	Part Time	1 (16.67%)	-	
	Casual	-	1 (16.67%)	
	Retired	-	1 (16.67%)	
	Illness/Disability	-	-	
Education	Primary Education	-	-	0.277
	High School	-	1 (16.67%)	
	Professional Training	2 (33.33%)	2 (33.33%)	
	Undergraduate	1 (16.67%)	3 (50.0%)	
	Postgraduate/Doctoral	3 (50.0%)	-	

Data presented as mean ± SD or N (%). Values of p < 0.05 are bolded. Abbreviations: BMI, body mass index; GWI, Gulf War Illness; HC, healthy controls.

Table 2 provides the WHODAS and FBC results compared between groups. There were no differences in FBC results between HC and GWI participants. Meanwhile, there were significant differences between HC and GWI results in communication and under-standing (p = 0.029), mobility (p = 0.007), self-care (p = 0.022), interpersonal connections (p = 0.024), life activities (p = 0.022), work participation (p = 0.007) and participation in society (p = 0.004). GWI participants had higher scores in all WHODAS domains, which indicates a significant increase in disability levels.

**Table 2:** Disability and full blood count results.

		HC	GWI	P-value
**WHODAS**				
Communication and Understanding		5.55 ± 8.19	28.47 ± 21.96	**0.029**
Mobility		0.0 ± 0.0	28.33 ± 21.13	**0.007**
Self-Care		0.0 ± 0.0	12.50 ± 16.77	**0.022**
Interpersonal Connections		3.12 ± 5.23	31.25 ± 20.16	**0.024**
Life Activities		0.0 ± 0.0	26.04 ± 22.51	**0.022**
Work Participation		0.0 ± 0.0	23.96 ± 18.72	**0.007**
Participation in Society		0.52 ± 1.28	37.50 ± 26.66	**0.004**
**Full blood count**				
White Cell Count (4.0–11.0 x10^9^/L)		5.90 ± 0.64	7.02 ± 1.69	0.109
Lymphocytes (1.0–4.0 x10^9^/L)		1.80 ± 0.58	1.78 ± 0.39	0.873
Neutrophils (2.0–8.0 x10^9^/L)		3.45 ± 0.88	4.46 ± 1.19	0.150
Monocytes (0.1–1.0 x10^9^/L)		0.42 ± 0.15	0.57 ± 0.18	0.054
Eosinophils (< 0.6 x10^9^/L)		0.17 ± 0.12	0.15 ± 0.11	0.376
Basophils (< 0.2 x10^9^/L)		0.04 ± 0.02	0.05 ± 0.03	0.373
Platelets (140–400 x10^9^/L)		227.5 ± 17.44	271.0 ± 81.39	0.261
Red Cell Count (3.8–5.2 x10^12^/L)		5.22 ± 0.29	5.06 ± 0.67	0.749
Haematocrit (0.33–0.47)		0.45 ± 0.03	0.45 ± 0.04	0.747
Haemoglobin (115–160 g/L)		154.7 ± 10.97	154.0 ± 16.89	0.873

Data presented as mean ± SD. Reference ranges for full blood count parameters have been included in the table. Values of p < 0.05 are bolded. Abbreviations: DAS, disability assessment schedule; GWI, Gulf War Illness; HC, healthy controls; WHO, World Health Organization.

Table 3 describes the symptoms experienced by GWI veterans in the month prior to blood donation, these symptoms might fluctuate over time as previously described [48]. All veterans reported fatigue, cognitive difficulties, pain and sleep disturbances, while respiratory disturbances were the less identified symptoms in this cohort, with only one patient referring respiratory problems. Furthermore, sensory, gastrointestinal and urinary disturbances were reported by 5 GWI participants (83.33%), while cardiovascular and immune symptoms by 4 GWI (66.66%). Thermostatic instabilities were reported by half of GWI participants. Interestingly, GWI participants from this study met ME/CFS clinical criteria, specifically all GWI met Canadian Consensus Criteria (CCC) for ME/CFS [49] and one GWI met the International Consensus Criteria (ICC) for ME/CFS [20].

**Table 3:** GWI participants symptom.

		GWI
Fatigue	Yes	6 (100.0%)
	No	0 (0.0%)
Cognitive Difficulties	Yes	6 (100.0%)
	No	0 (0.0%)
Pain	Yes	6 (100.0%)
	No	0 (0.0%)
Sleep Disturbances	Yes	6 (100.0%)
	No	0 (0.0%)
Sensory Disturbances	Yes	5 (83.33%)
	No	1 (16.67%)
Immune Disturbances	Yes	4 (66.66%)
	No	2 (33.34%)
Gastrointestinal Disturbances	Yes	5 (83.33%)
	No	1 (16.67%)
Cardiovascular Disturbances	Yes	4 (66.66%)
	No	2 (33.34%)
Respiratory Disturbances	Yes	1 (16.67%)
	No	5 (83.33%)
Thermostatic Instability	Yes	3 (50.0%)
	No	3 (50.0%)
Urinary Disturbances	Yes	5 (83.33%)
	No	1 (16.67%)

Data presented as mean ± SD and N (%). Abbreviations: GWI, Gulf War Illness.

### Electrophysiological experiments

**Fig 1:**
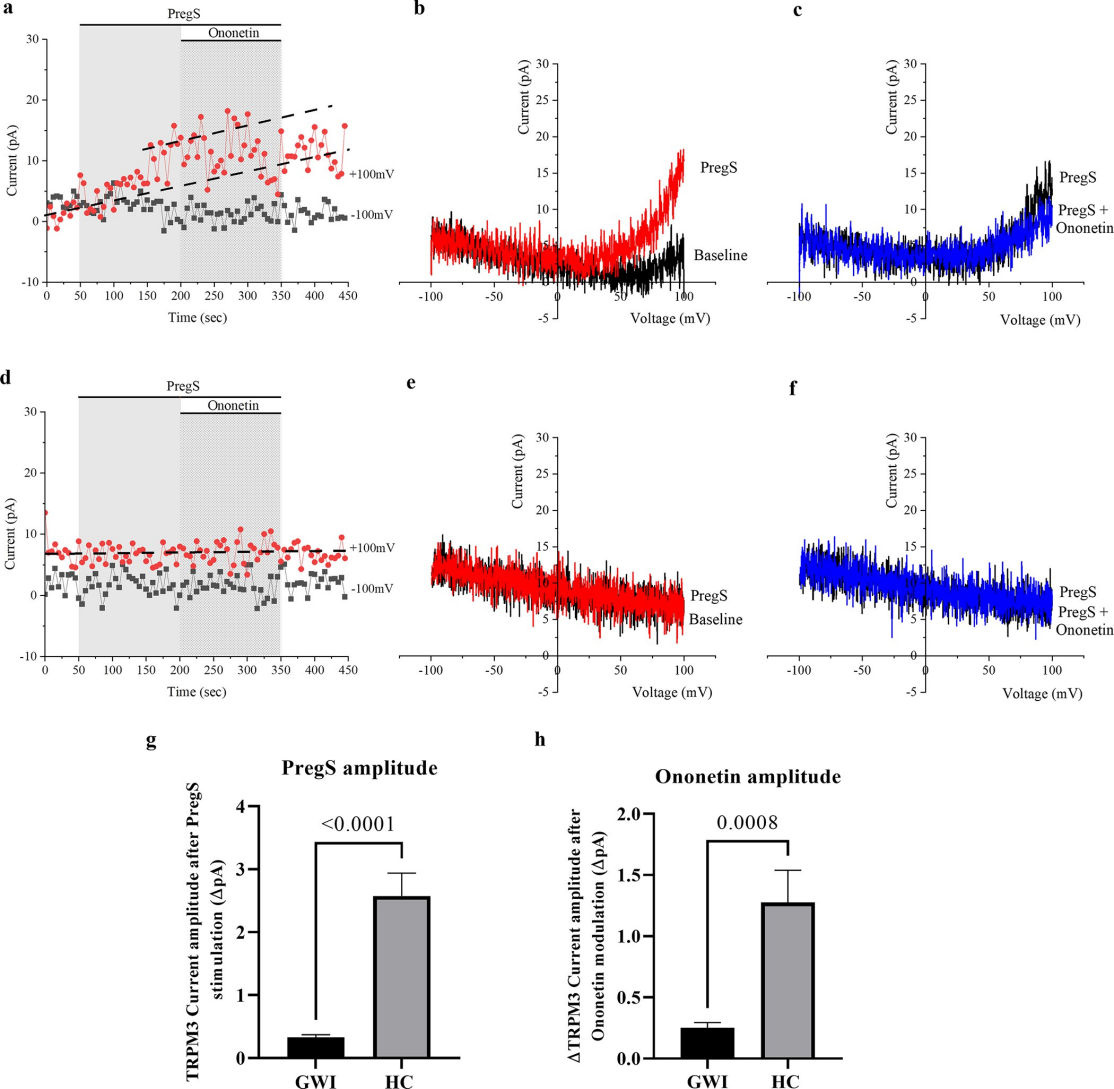
Representation of TRPM3 ion channel activity in NK cells from HC and GWI participants (whole-cell patch-clamp). a, b, c—Current obtained in whole-cell patch-clamp condition in a NK cell from a HC participant. (a) A representative time-series of current amplitude at +100 mV and −100 mV. (b) I–V curve before and after 100 μΜ PregS stimulation. (c) I–V curve on PregS stimulation and after modulation with 10 μΜ Ononetin in the presence of PregS. d, e, f—Current obtained in whole-cell patch-clamp condition in a NK cell from a GWI participant. (d) A representative time-series of current amplitude at +100 mV and −100 mV. (e) I–V curve before and after 100 μΜ PregS stimulation. (f) I–V curve on PregS stimulation and after modulation with 10 μΜ Ononetin in the presence of PregS. g–h: Bar graphs representing TRPM3 current amplitude at +100 mV, (g) is regarding TRPM3 stimulation with 100 μΜ PregS in GWI (N = 6; n = 47) compared with HC participants (N = 6; n = 48), while (h) is regarding TRPM3 modulation with 10 μΜ Ononetin in the presence of PregS in GWI (N = 6; n = 42) compared with HC participants (N = 6; n = 39). Dash-lines in time-series illustrate the baseline and PregS effects. N refers to number of participants and n to number of records analysed. Data presented as mean ± SEM and determined by Mann-Whitney U test. Abbreviations: GWI, Gulf War Illness; HC, healthy control; NK, natural killer; PregS, pregnenolone sulfate.

The gold standard patch-clamp technique for studying ion channels was performed to characterize TRPM3 using whole-cell configuration in this study. In line with extensive literature [26,50–55], the TRPM3 agonist pregnenolone sulfate (PregS) activates these ion channels inducing an increase in intracellular Ca^2+^ concentration in HC cells, as represented in Fig 1A and 1B. Under voltage-clamp conditions, 100 μM PregS induced small outward rectifying currents in most NK cells isolated from HC which showed characteristic TRPM3 current–voltage relationship (I–V) (Fig 1B). However, in NK cells from the GWI group, the application of 100 μM of PregS stimulated only a few NK cells. To statistically compare TRPM3 ion channel function between both groups, amplitudes were determined for each recording as a change in amplitude from baseline to PregS induced peak, as represented in time-series graphs (Fig 1A and 1D). In this investigation a significantly smaller amplitude of PregS-evoked currents was found in NK cells isolated from GWI participants in comparison to cells from HC (Fig 1G, p < 0.0001), a result that indicates people diagnosed GWI have TRPM3 impaired function. Fig 1 provides examples of recordings in a NK cell from a HC (Fig 1A–1C) and GWI participant (Fig 1D–1F).

**Fig 2:**
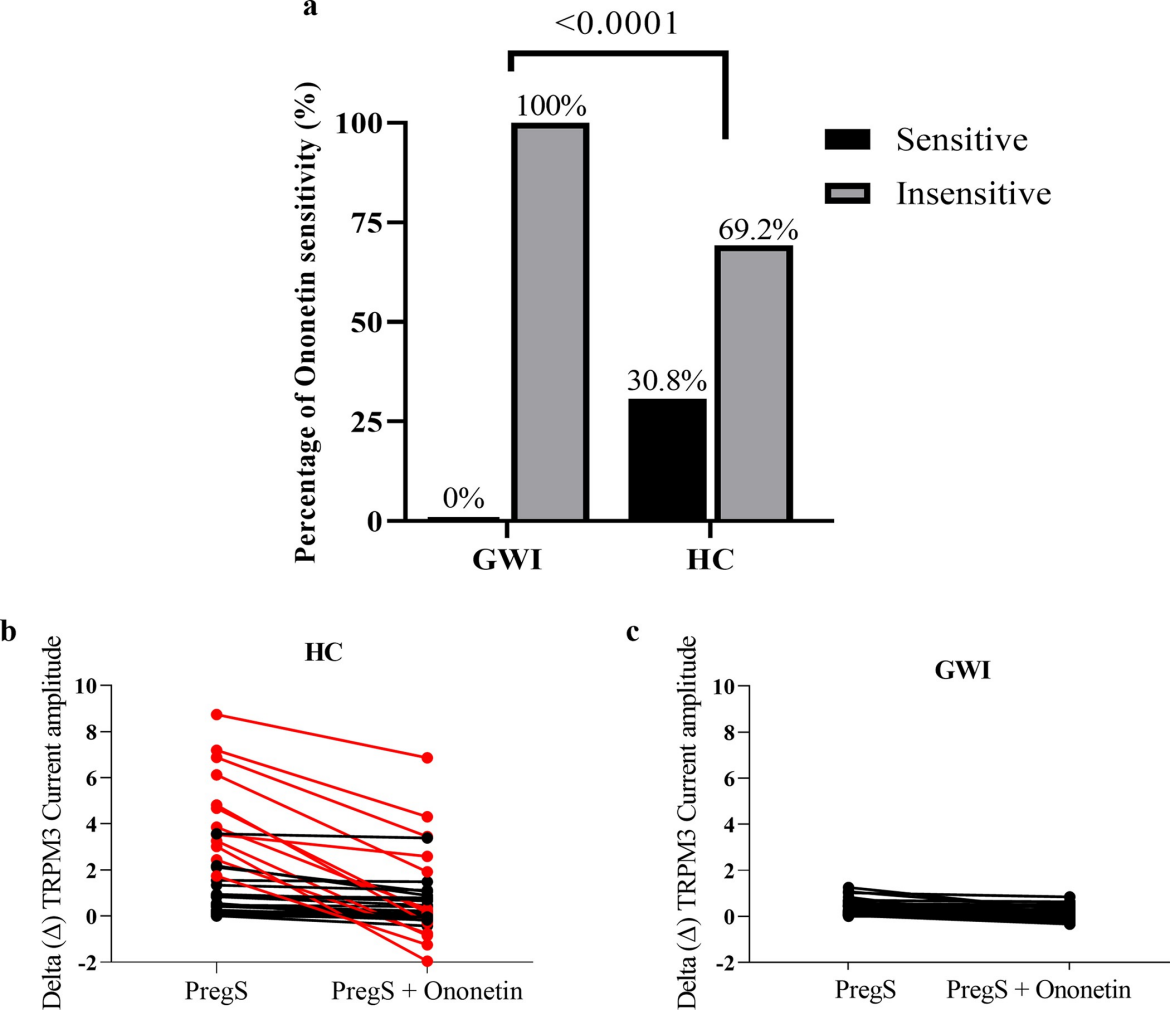
Ononetin results. (a) Bar graphs representing percentage of sensitive and insensitive NK cells to 10 μΜ Ononetin in presence of PregS, from HC (N = 6; n = 39), and GWI (N = 6; n = 42). (b—c) Scatter plots representing change of each current amplitude before and after application of Ononetin in presence of PregS in NK cells from HC and GWI participants respectively. Each red line represented a cell sensitive to Ononetin as a reduction in amplitude was recorded. N to number of participants and n to number of records analysed. Abbreviations: GWI, Gulf War Illness; HC, healthy control; NK, natural killer; PregS, pregnenolone sulfate.

To confirm the presence of TRPM3, 10 μM of the antagonist Ononetin was applied in the presence of PregS whereby a reduction in ionic currents indicated sensitivity to Ononetin and consequently the presence of TRPM3 [56]. As expected, PregS-evoked ionic currents were successfully suppressed during Ononetin application in NK cells from HCs (Fig 1A) and an outward rectification I-V curve was observed (Fig 1C). However, there was a significant reduction in Ononetin amplitude in currents obtained in NK cells from GWI patients compared to HC individuals (Fig 1H, p = 0.0008). In addition, there was a significant reduction in the number of NK cells from GWI participants sensitive to Ononetin compared with HC (30.8%, Fig 2A p < 0.0001). In Fig 2, scatter plots demonstrate each current amplitude with PregS and Ononetin modulation, to show changes during patch-clamp protocol in NK cells from HC (Fig 2B) and GWI (Fig 2C). These results confirmed the involvement of TRPM3 ion channels in PregS-evoked currents in NK cells isolated from HC and the TRPM3 dysfunction in cells from GWI participants.

## Discussion

Our data is the first to report significant impairment in TRPM3 ion channel function in NK cells from participants with GWI compared to HC. TRPM3 is a non-selective cation channel that highly exhibits permeability to Ca^2+^ and plays a critical role in a variety of biological processes due to its contribution in the Ca^2+^ signalling regulation [24,57,58]. For instance, Ca^2+^ is an essential element for intracellular signalling pathways, immunity activation, proliferation and maintenance of cellular activities, degranulation, release of cytolytic proteins and homeostasis [24,28,37,57–60]. Likewise, abnormal intracellular Ca^2+^ concentration associated with dysfunctional TRPM3 may cause inadequate cell function and imbalance intracellular signalling pathways [26,27,61].

The design of the present study used a primary NK cell model to investigate TRPM3 function as the immune system plays a substantial role in GWI. Many studies have supported impaired immunological function and inflammation as significant components of GWI pathophysiology [23,62,63]. Whistler and colleagues results showed significant reduction in NK cell cytotoxicity which is indicative of immune disruption in GWI patients [23]. TRP channels are involved in oxidative stress, pain and inflammatory responses, while TRPM3 specifically in peripheral nociceptors have pronociceptive and pro-inflammatory properties [37,64,65]. Elhaj et al recently identified an increase in interleukin 6 (IL-6) and C-reactive protein (CRP) levels in GWI patients compared with other veterans without GWI, which is consistent with previous research [62,66] and corroborate the role of chronic neuroimmune and neuroinflammation disturbances [1].

Recent literature in the immune field, has described similarities among individuals with GWI, long COVID-19 or ME/CFS, including symptoms, impact on quality of life, lack of diagnostic testing and treatment [6]Click or tap here to enter text. Notably, our present finding of decreased TRPM3 function in NK cells from GWI is compatible with results reported in ME/CFS and long COVID cohorts [26,61,67], suggesting a common overlap of TRPM3 dysfunction, or TRP ion channels, in the pathomechanism of each of these diseases. Support for this argument is highlighted by TRP ion channels being modulated by numerous stimuli, for example, by environmental (e.g. temperature, light, chemicals), mechanical (e.g. osmolarity, pressure), natural (e.g. herbs or spices, venoms, toxins), endogenous factors, tissue damage, infection and inflammation [40,41,68–71]. In addition, people diagnosed with these diseases report having been exposed to some of these threats/stimuli prior to the symptom’s onset. For instance, the precedent factor for long COVID-19 is unequivocally the Severe Acute Respiratory Syndrome Coronavirus 2 (SARS-CoV-2) infection, while ME/CFS patients often report prior infections, stressors, trauma and toxin exposure, with about 75% of cases associated to infection-like episodes before ME/CFS onset [22,72]. The hypothesis of TRP ion channel dysfunction underpinning the potential pathomechanism due to the intense exposure of a wide variety of chemical components, as well as vaccine and prophylactic drugs, is highly plausible [5,6,9,17].

Furthermore, TRP ion channels have been recognized as potentially associated with multisystem diseases and emerging as important drug targets due to their ubiquitous expression in cells from human organs and peripheral tissues [30,40,41,44,73,74]. Fonfria et al. characterized the expression of all TRPM family members in many human tissues, TRPM3 specifically was detected in cells from the brain, pituitary, kidney, adipose tissue, pancreas, prostate, and bone [75]. Other researchers also reported TRPM3 in different parts of the central nervous and cardiovascular systems, immune cells, liver, urinary and genital systems [24,26,50,73,76,77]. Despite the present study having assessed only TRPM3 ion channels in NK cells, these findings suggest this ion channel is dysfunctional in other tissue cells in GWI participants. Hence, the ubiquitous expression of TRPM3 ion channels is compatible with the constellation of GWI symptoms.

Interestingly, animal studies have reported that other TRP channels are related to chemical exposure suffered by veterans in the GW that could potentially explain their symptoms. For instance, Ding et al demonstrated through application of Transient Receptor Potential Ankyrin 1 (TRPA1) antagonists that TRPA1 is a principal mediator of organophosphate-induced delayed neuropathy, a condition that occurs due to acute or chronic exposure and is usually correlated to acetylcholinesterase suppression [78]. However, Ding and colleagues reported that tested organophosphates activated TRPA1 (also a Ca^2+^ permeable non-selective cation channel), increased the influx of Ca^2+^ while TRPA1 antagonists significantly relieved organophosphate-induced neuropathy models [78].

A substantial variety of animal models have been developed to investigate GWI pathology, mainly induced by animal exposure to a single or combination of threats/agents (organophosphates, PB, stressors) to reproduce the GW environment [9]. Although animal studies are crucial tools for health research, results should not be extrapolated to humans. Principal limitations of animal models are genomic differences that studies evaluate on short term effects disregarding decades of GWI, and focusing on symptoms individually [1,77,79]. In contrast, our results are directly based on freshly isolated immune cells from veterans diagnosed with GWI compared to cells from HC. Moreover, these results were obtained evaluating endogenous TRPM3 from human primary cells freshly isolated from blood, through the gold standard technique for studying ion channels.

Notably, the importance of identifying TRPM3 as a biomarker for GWI is mainly to facilitate a diagnosis. Currently, due to the absence of a screening or specific diagnostic test, GWI remains diagnosed through case definitions and exclusion of other conditions that would not explain the symptoms expressed by GW veterans [80]. Nevertheless, the novel findings of these studies provide the perspective of developing a test focused on the characterization of ion channel function using an easily acquired biological sample.

## Conclusion

In conclusion, the present study creates a rationale for future studies analysing other TRP channels in plasma membrane, as well as organelles. It further provides an opportunity to lead investigations of therapeutic strategies to treat and manage the GWI condition, to alleviate severe symptoms and consequently improve their quality of life. Further, ion channel studies are necessary to investigate the contribution of other TRP channels in the pathophysiology of GWI.

## Supporting information

S1 Fig NK cell purity. NK cell purity was acquired at 10,000 events using the BD LSRFortessa^TM^ X-20. NK cells, defined as CD3^-^CD56^+^ lymphocytes were 96.08% ± 0.953 for HC and 94.33% ± 1.144 for participants with GWI. (a) lymphocytes were gated based on Side Scatter (SSC) and versus Forward Scatter (FSC). (b) CD3^-^ cell population was gated from selected lymphocyte population. Gating was identified through isotype controls. (c) NK cell purity was based on CD56^+^ population from the CD3^-^ population. (d) Bar graphs illustrating percentage of NK cell population. Data presented as mean ± SEM and determined by Mann-Whitney U test. Abbreviation: GWI, Gulf War Illness; HC, healthy controls; NK, natural killer.
(TIF)

S2 Fig Individual recording analysis. Two representative time-series of current amplitude at +100 mV and −100 mV showing the effect of PregS and Ononetin in the presence of PregS. Baseline = blue dash-lines; PregS baseline = green dash-lines. (A) Baseline; (B) PregS points; (C) PregS baseline; (D) Ononetin points. PregS amplitude = (B)–(A) and Ononetin amplitude = (C)–(D). PregS was effective when presented with an increase at +100 mV current and Ononetin was effective when there is a decrease at +100 mV current. No difference means drugs were not effective to stimulate agonist or antagonist effect on TRPM3 ion channels. On (a), PregS and Ononetin in the presence of PregS were effective, however, on (b) only PregS was effective.
(TIF)

## References

[1] Elhaj R, Reynolds JM. Chemical exposures and suspected impact on Gulf War Veterans. Mil Med Res. 2023;10(1):11. Epub 20230308. doi: 10.1186/s40779-023-00449-9 ; PubMed Central PMCID: PMC9993698. 36882803 PMC9993698

[2] Gean EG, Ayers CK, Winchell KA, Freeman M, Press AM, Paynter R, et al. Biological measures and diagnostic tools for Gulf War Illness—A systematic review. Life Sci. 2021;275:119360. Epub 20210316. doi: 10.1016/j.lfs.2021.119360 . 33741418

[3] Institute of Medicine. In: Hernandez LM, Durch JS, Blazer DG II, Hoverman IV, editors. Gulf War Veterans: Measuring Health. Washington (DC): The National Academies Press; 1999. 25077231

[4] National Academies of Sciences, Engineering, and Medicine,. In: Cory-Slechta D, Wedge R, editors. Gulf War and Health: Volume 10: Update of Health Effects of Serving in the Gulf War, 2016. Washington (DC) 2016. 27054224

[5] Hernandez S, Morales-Soto W, Grubisic V, Fried D, Gulbransen BD. Pyridostigmine bromide exposure creates chronic, underlying neuroimmune disruption in the gastrointestinal tract and brain that alters responses to palmitoylethanolamide in a mouse model of Gulf War Illness. Neuropharmacology. 2020;179:108264. Epub 20200803. doi: 10.1016/j.neuropharm.2020.108264 ; PubMed Central PMCID: PMC7572863. 32758565 PMC7572863

[6] James LM, Georgopoulos AP. At the Root of 3 "Long" Diseases: Persistent Antigens Inflicting Chronic Damage on the Brain and Other Organs in Gulf War Illness, Long-COVID-19, and Chronic Fatigue Syndrome. Neurosci Insights. 2022;17:26331055221114817. Epub 20220722. doi: 10.1177/26331055221114817 ; PubMed Central PMCID: PMC9335483. 35910083 PMC9335483

[7] Iversen A, Chalder T, Wessely S. Gulf War Illness: lessons from medically unexplained symptoms. Clin Psychol Rev. 2007;27(7):842–54. Epub 20070717. doi: 10.1016/j.cpr.2007.07.006 . 17707114

[8] Committee on Health Effects Associated with Exposures During the Gulf War, Division of Health, Promotion and Disease Prevention. In: Fulco CE, Liverman CT, Sox HC, editors. Gulf War and Health: Volume 1 Depleted Uranium, Sarin, Pyridostigmine Bromide, Vaccines. Washington (DC) 2000. 25057724

[9] White RF, Steele L, O’Callaghan JP, Sullivan K, Binns JH, Golomb BA, et al. Recent research on Gulf War illness and other health problems in veterans of the 1991 Gulf War: Effects of toxicant exposures during deployment. Cortex. 2016;74:449–75. Epub 20150925. doi: 10.1016/j.cortex.2015.08.022 ; PubMed Central PMCID: PMC4724528. 26493934 PMC4724528

[10] Moffett K, Crosson B, Spence JS, Case K, Levy I, Gopinath K, et al. Word-finding impairment in veterans of the 1991 Persian Gulf War. Brain Cogn. 2015;98:65–73. Epub 20150623. doi: 10.1016/j.bandc.2015.05.005 . 26114921

[11] Cohen DE, Sullivan KA, McNeil RB, Gulf War Illness Common Data Elements Working G, Symptoms Assessment Working G, McNeil RB, et al. A common language for Gulf War Illness (GWI) research studies: GWI common data elements. Life Sci. 2022;290:119818. Epub 20210802. doi: 10.1016/j.lfs.2021.119818 ; PubMed Central PMCID: PMC9267452. 34352259 PMC9267452

[12] Baraniuk JN. Review of the Midbrain Ascending Arousal Network Nuclei and Implications for Myalgic Encephalomyelitis/Chronic Fatigue Syndrome (ME/CFS), Gulf War Illness (GWI) and Postexertional Malaise (PEM). Brain Sci. 2022;12(2). Epub 20220119. doi: 10.3390/brainsci12020132 ; PubMed Central PMCID: PMC8870178. 35203896 PMC8870178

[13] Lindheimer JB, Stegner AJ, Wylie GR, Klein-Adams JC, Almassi NE, Ninneman JV, et al. Post-exertional malaise in veterans with gulf war illness. Int J Psychophysiol. 2020;147:202–12. Epub 20191128. doi: 10.1016/j.ijpsycho.2019.11.008 ; PubMed Central PMCID: PMC6957714. 31786249 PMC6957714

[14] Fukuda K, Nisenbaum R, Stewart G, Thompson WW, Robin L, Washko RM, et al. Chronic multisymptom illness affecting Air Force veterans of the Gulf War. JAMA. 1998;280(11):981–8. doi: 10.1001/jama.280.11.981 . 9749480

[15] Steele L. Prevalence and patterns of Gulf War illness in Kansas veterans: association of symptoms with characteristics of person, place, and time of military service. Am J Epidemiol. 2000;152(10):992–1002. doi: 10.1093/aje/152.10.992 . 11092441

[16] Macht VA, Woodruff JL, Maissy ES, Grillo CA, Wilson MA, Fadel JR, et al. Pyridostigmine bromide and stress interact to impact immune function, cholinergic neurochemistry and behavior in a rat model of Gulf War Illness. Brain Behav Immun. 2019;80:384–93. Epub 20190403. doi: 10.1016/j.bbi.2019.04.015 ; PubMed Central PMCID: PMC6790976. 30953774 PMC6790976

[17] Ojo JO, Abdullah L, Evans J, Reed JM, Montague H, Mullan MJ, et al. Exposure to an organophosphate pesticide, individually or in combination with other Gulf War agents, impairs synaptic integrity and neuronal differentiation, and is accompanied by subtle microvascular injury in a mouse model of Gulf War agent exposure. Neuropathology. 2014;34(2):109–27. Epub 20130930. doi: 10.1111/neup.12061 . 24118348

[18] Rayhan RU, Ravindran MK, Baraniuk JN. Migraine in gulf war illness and chronic fatigue syndrome: prevalence, potential mechanisms, and evaluation. Front Physiol. 2013;4:181. Epub 20130724. doi: 10.3389/fphys.2013.00181 ; PubMed Central PMCID: PMC3721020. 23898301 PMC3721020

[19] Keating JA, Shaughnessy C, Baubie K, Kates AE, Putman-Buehler N, Watson L, et al. Characterising the gut microbiome in veterans with Gulf War Illness: a protocol for a longitudinal, prospective cohort study. BMJ Open. 2019;9(8):e031114. Epub 20190819. doi: 10.1136/bmjopen-2019-031114 ; PubMed Central PMCID: PMC6707676. 31431446 PMC6707676

[20] Carruthers BM, van de Sande MI, De Meirleir KL, Klimas NG, Broderick G, Mitchell T, et al. Myalgic encephalomyelitis: International Consensus Criteria. J Intern Med. 2011;270(4):327–38. Epub 2011/07/23. doi: 10.1111/j.1365-2796.2011.02428.x ; PubMed Central PMCID: PMC3427890. 21777306 PMC3427890

[21] Marshall-Gradisnik S, Eaton-Fitch N. Understanding myalgic encephalomyelitis. Science. 2022;377(6611):1150–1. Epub 2022/09/09. doi: 10.1126/science.abo1261 . 36074854

[22] Choutka J, Jansari V, Hornig M, Iwasaki A. Unexplained post-acute infection syndromes. Nat Med. 2022;28(5):911–23.Epub 2022/05/19. doi: 10.1038/s41591-022-01810-6 . 35585196

[23] Whistler T, Fletcher MA, Lonergan W, Zeng XR, Lin JM, Laperriere A, et al. Impaired immune function in Gulf War Illness. BMC Med Genomics. 2009;2:12. Epub 20090305. doi: 10.1186/1755-8794-2-12 ; PubMed Central PMCID: PMC2657162. 19265525 PMC2657162

[24] Nguyen T, Staines D, Nilius B, Smith P, Marshall-Gradisnik S. Novel identification and characterisation of Transient receptor potential melastatin 3 ion channels on Natural Killer cells and B lymphocytes: effects on cell signalling in Chronic fatigue syndrome/Myalgic encephalomyelitis patients. Biol Res. 2016;49(1):27. Epub 2016/06/02. doi: 10.1186/s40659-016-0087-2 ; PubMed Central PMCID: PMC4888729. 27245705 PMC4888729

[25] Du Preez S, Eaton-Fitch N, Cabanas H, Staines D, Marshall-Gradisnik S. Characterization of IL-2 Stimulation and TRPM7 Pharmacomodulation in NK Cell Cytotoxicity and Channel Co-Localization with PIP2 in Myalgic Encephalomyelitis/Chronic Fatigue Syndrome Patients. Int J Environ Res Public Health. 2021;18(22). Epub 2021/11/28. doi: 10.3390/ijerph182211879 ; PubMed Central PMCID: PMC8618557. 34831634 PMC8618557

[26] Cabanas H, Muraki K, Eaton N, Balinas C, Staines D, Marshall-Gradisnik S. Loss of Transient Receptor Potential Melastatin 3 ion channel function in natural killer cells from Chronic Fatigue Syndrome/Myalgic Encephalomyelitis patients. Mol Med. 2018;24(1):44. Epub 2018/08/24. doi: 10.1186/s10020-018-0046-1 ; PubMed Central PMCID: PMC6092868. 30134818 PMC6092868

[27] Eaton-Fitch N, Du Preez S, Cabanas H, Muraki K, Staines D, Marshall-Gradisnik S. Impaired TRPM3-dependent calcium influx and restoration using Naltrexone in natural killer cells of myalgic encephalomyelitis/chronic fatigue syndrome patients. J Transl Med. 2022;20(1):94. Epub 2022/02/18. doi: 10.1186/s12967-022-03297-8 ; PubMed Central PMCID: PMC8848670. 35172836 PMC8848670

[28] Balinas C, Cabanas H, Staines D, Marshall-Gradisnik S. Transient receptor potential melastatin 2 channels are overexpressed in myalgic encephalomyelitis/chronic fatigue syndrome patients. J Transl Med. 2019;17(1):401. Epub 2019/12/05. doi: 10.1186/s12967-019-02155-4 ; PubMed Central PMCID: PMC6891975. 31796045 PMC6891975

[29] Cabanas H, Muraki K, Eaton-Fitch N, Staines DR, Marshall-Gradisnik S. Potential Therapeutic Benefit of Low Dose Naltrexone in Myalgic Encephalomyelitis/Chronic Fatigue Syndrome: Role of Transient Receptor Potential Melastatin 3 Ion Channels in Pathophysiology and Treatment. Front Immunol. 2021;12:687806. Epub 2021/07/31. doi: 10.3389/fimmu.2021.687806 ; PubMed Central PMCID: PMC8313851. 34326841 PMC8313851

[30] Ramsey IS, Delling M, Clapham DE. An introduction to TRP channels. Annu Rev Physiol. 2006;68:619–47. Epub 2006/02/08. doi: 10.1146/annurev.physiol.68.040204.100431 . 16460286

[31] Wu LJ, Sweet TB, Clapham DE. International Union of Basic and Clinical Pharmacology. LXXVI. Current progress in the mammalian TRP ion channel family. Pharmacol Rev. 2010;62(3):381–404. Epub 2010/08/19. doi: 10.1124/pr.110.002725 ; PubMed Central PMCID: PMC2964900. 20716668 PMC2964900

[32] Moran M, McAlexander M, Bíró T, Szallasi A. Transient receptor potential channels as therapeutic targets. Nat Rev Drug Discov. 2011. doi: 10.1038/nrd3456 21804597

[33] Salgado VL. Insect TRP channels as targets for insecticides and repellents. J Pestic Sci. 2017;42(1):1–6. doi: 10.1584/jpestics.D16-104 ; PubMed Central PMCID: PMC6140660. 30363111 PMC6140660

[34] Voets T, Talavera K, Owsianik G, Nilius B. Sensing with TRP channels. Nat Chem Biol. 2005;1(2):85–92. doi: 10.1038/nchembio0705-85 . 16408004

[35] Nilius B, Talavera K, Owsianik G, Prenen J, Droogmans G, Voets T. Gating of TRP channels: a voltage connection? J Physiol. 2005;567(Pt 1):35–44. Epub 20050505. doi: 10.1113/jphysiol.2005.088377 ; PubMed Central PMCID: PMC1474154. 15878939 PMC1474154

[36] Nilius B, Owsianik G. The transient receptor potential family of ion channels. Genome Biol. 2011;12(3):218. Epub 2011/03/16. doi: 10.1186/gb-2011-12-3-218 ; PubMed Central PMCID: PMC3129667. 21401968 PMC3129667

[37] Hasan R, Zhang X. Ca(2+) Regulation of TRP Ion Channels. Int J Mol Sci. 2018;19(4). Epub 2018/04/25. doi: 10.3390/ijms19041256 ; PubMed Central PMCID: PMC5979445. 29690581 PMC5979445

[38] Li H. TRP Channel Classification. Adv Exp Med Biol. 2017;976:1–8. Epub 2017/05/17. doi: 10.1007/978-94-024-1088-4_1 . 28508308

[39] Clement D, Goodridge JP, Grimm C, Patel S, Malmberg KJ. TRP Channels as Interior Designers: Remodeling the Endolysosomal Compartment in Natural Killer Cells. Front Immunol. 2020;11:753. Epub 2020/05/16. doi: 10.3389/fimmu.2020.00753 ; PubMed Central PMCID: PMC7198808. 32411146 PMC7198808

[40] Parenti A, De Logu F, Geppetti P, Benemei S. What is the evidence for the role of TRP channels in inflammatory and immune cells? Br J Pharmacol. 2016;173(6):953–69. Epub 2015/11/26. doi: 10.1111/bph.13392 ; PubMed Central PMCID: PMC5341240. 26603538 PMC5341240

[41] Rather MA, Khan A, Wang L, Jahan S, Rehman MU, Makeen HA, et al. TRP channels: Role in neurodegenerative diseases and therapeutic targets. Heliyon. 2023;9(6):e16910. Epub 20230602. doi: 10.1016/j.heliyon.2023.e16910 ; PubMed Central PMCID: PMC10272313. 37332910 PMC10272313

[42] Himmel NJ, Cox DN. Transient receptor potential channels: current perspectives on evolution, structure, function and nomenclature. Proc Biol Sci. 2020;287(1933):20201309. Epub 2020/08/28. doi: 10.1098/rspb.2020.1309 ; PubMed Central PMCID: PMC7482286. 32842926 PMC7482286

[43] Santoni G, Farfariello V, Liberati S, Morelli MB, Nabissi M, Santoni M, et al. The role of transient receptor potential vanilloid type-2 ion channels in innate and adaptive immune responses. Front Immunol. 2013;4:34. Epub 2013/02/20. doi: 10.3389/fimmu.2013.00034 ; PubMed Central PMCID: PMC3572502. 23420671 PMC3572502

[44] Froghi S, Grant CR, Tandon R, Quaglia A, Davidson B, Fuller B. New Insights on the Role of TRP Channels in Calcium Signalling and Immunomodulation: Review of Pathways and Implications for Clinical Practice. Clin Rev Allergy Immunol. 2021;60(2):271–92. Epub 2021/01/07. doi: 10.1007/s12016-020-08824-3 ; PubMed Central PMCID: PMC7985118. 33405100 PMC7985118

[45] Hong C, Jeong B, Park HJ, Chung JY, Lee JE, Kim J, et al. TRP Channels as Emerging Therapeutic Targets for Neurodegenerative Diseases. Front Physiol. 2020;11:238. Epub 20200415. doi: 10.3389/fphys.2020.00238 ; PubMed Central PMCID: PMC7174697. 32351395 PMC7174697

[46] Üstün T, Kostanjsek N, Chatterji S, Rehm J. Measuring health and disability: Manual for WHO disability assessment schedule—WHODAS 2.0.2010 2022 Mar 28. Available from: Available from: https://apps.who.int/iris/handle/10665/43974.

[47] Andrews G, Kemp A, Sunderland M, Von Korff M, Ustun TB. Normative data for the 12 item WHO Disability Assessment Schedule 2.0. PLoS One. 2009;17(12):e8343. doi: 10.1371/journal.pone.0008343 20020047 PMC2791224

[48] Zundel CG, Heeren T, Grasso CM, Spiro A 3rd, Proctor SP, Sullivan K, et al. Changes in Health Status in the Ft. Devens Gulf War Veterans Cohort: 1997–2017. Neurosci Insights. 2020;15:2633105520952675. Epub 20200820. doi: 10.1177/2633105520952675 ; PubMed Central PMCID: PMC7444112. 32914090 PMC7444112

[49] Carruthers BM, Jain AK, De Meirleir KL, Peterson DL, Klimas NG, Lerner A, et al. Myalgic encephalomyelitis/chronic fatigue syndrome: clinical working case definition, diagnostic and treatment protocols. Journal of Chronic Fatigue Syndrome. 2003;11:7–115.

[50] Naylor J, Li J, Milligan CJ, Zeng F, Sukumar P, Hou B, et al. Pregnenolone sulphate- and cholesterol-regulated TRPM3 channels coupled to vascular smooth muscle secretion and contraction. Circ Res. 2010;106(9):1507–15. Epub 2010/04/03. doi: 10.1161/CIRCRESAHA.110.219329 ; PubMed Central PMCID: PMC2877666. 20360246 PMC2877666

[51] Majeed Y, Agarwal AK, Naylor J, Seymour VA, Jiang S, Muraki K, et al. Cis-isomerism and other chemical requirements of steroidal agonists and partial agonists acting at TRPM3 channels. Br J Pharmacol. 2010;161(2):430–41. Epub 2010/08/26. doi: 10.1111/j.1476-5381.2010.00892.x ; PubMed Central PMCID: PMC2989593. 20735426 PMC2989593

[52] Persoons E, Kerselaers S, Voets T, Vriens J, Held K. Partial Agonistic Actions of Sex Hormone Steroids on TRPM3 Function. Int J Mol Sci. 2021;22(24). Epub 2021/12/25. doi: 10.3390/ijms222413652 ; PubMed Central PMCID: PMC8708174. 34948452 PMC8708174

[53] Wagner TF, Loch S, Lambert S, Straub I, Mannebach S, Mathar I, et al. Transient receptor potential M3 channels are ionotropic steroid receptors in pancreatic beta cells. Nat Cell Biol. 2008;10(12):1421–30. Epub 2008/11/04. doi: 10.1038/ncb1801 . 18978782

[54] Vanneste M, Mulier M, Nogueira Freitas AC, Van Ranst N, Kerstens A, Voets T, et al. TRPM3 Is Expressed in Afferent Bladder Neurons and Is Upregulated during Bladder Inflammation. Int J Mol Sci. 2021;23(1). Epub 20211222. doi: 10.3390/ijms23010107 ; PubMed Central PMCID: PMC8745475. 35008533 PMC8745475

[55] Alonso-Carbajo L, Alpizar YA, Startek JB, Lopez-Lopez JR, Perez-Garcia MT, Talavera K. Activation of the cation channel TRPM3 in perivascular nerves induces vasodilation of resistance arteries. J Mol Cell Cardiol. 2019;129:219–30. Epub 20190307. doi: 10.1016/j.yjmcc.2019.03.003 . 30853321

[56] Straub I, Mohr F, Stab J, Konrad M, Philipp SE, Oberwinkler J, et al. Citrus fruit and fabacea secondary metabolites potently and selectively block TRPM3. Br J Pharmacol. 2013;168(8):1835–50. Epub 2012/11/30. doi: 10.1111/bph.12076 ; PubMed Central PMCID: PMC3623054. 23190005 PMC3623054

[57] Schwarz EC, Qu B, Hoth M. Calcium, cancer and killing: the role of calcium in killing cancer cells by cytotoxic T lymphocytes and natural killer cells. Biochim Biophys Acta. 2013;1833(7):1603–11. Epub 2012/12/12. doi: 10.1016/j.bbamcr.2012.11.016 . 23220009

[58] Clapham DE. Calcium signaling. Cell. 2007;131(6):1047–58. Epub 2007/12/18. doi: 10.1016/j.cell.2007.11.028 . 18083096

[59] Panyi G, Varga Z, Gaspar R. Ion channels and lymphocyte activation. Immunol Lett. 2004;92(1–2):55–66. doi: 10.1016/j.imlet.2003.11.020 . 15081528

[60] Zhou X, Friedmann KS, Lyrmann H, Zhou Y, Schoppmeyer R, Knorck A, et al. A calcium optimum for cytotoxic T lymphocyte and natural killer cell cytotoxicity. J Physiol. 2018;596(14):2681–98. Epub 20180312. doi: 10.1113/JP274964 ; PubMed Central PMCID: PMC6046087. 29368348 PMC6046087

[61] Cabanas H, Muraki K, Balinas C, Eaton-Fitch N, Staines D, Marshall-Gradisnik S. Validation of impaired Transient Receptor Potential Melastatin 3 ion channel activity in natural killer cells from Chronic Fatigue Syndrome/ Myalgic Encephalomyelitis patients. Mol Med. 2019;25(1):14. Epub 2019/04/25. doi: 10.1186/s10020-019-0083-4 ; PubMed Central PMCID: PMC6480905. 31014226 PMC6480905

[62] Johnson GJ, Slater BC, Leis LA, Rector TS, Bach RR. Blood Biomarkers of Chronic Inflammation in Gulf War Illness. PLoS One. 2016;11(6):e0157855. Epub 20160628. doi: 10.1371/journal.pone.0157855 ; PubMed Central PMCID: PMC4924830. 27352030 PMC4924830

[63] Trageser KJ, Sebastian-Valverde M, Naughton SX, Pasinetti GM. The Innate Immune System and Inflammatory Priming: Potential Mechanistic Factors in Mood Disorders and Gulf War Illness. Front Psychiatry. 2020;11:704. Epub 20200723. doi: 10.3389/fpsyt.2020.00704 ; PubMed Central PMCID: PMC7396635. 32848904 PMC7396635

[64] Dembla S, Behrendt M, Mohr F, Goecke C, Sondermann J, Schneider FM, et al. Anti-nociceptive action of peripheral mu-opioid receptors by G-beta-gamma protein-mediated inhibition of TRPM3 channels. Elife. 2017;6. Epub 2017/08/23. doi: 10.7554/eLife.26280 ; PubMed Central PMCID: PMC5593507. 28826482 PMC5593507

[65] Huang Y, Fliegert R, Guse AH, Lu W, Du J. A structural overview of the ion channels of the TRPM family. Cell Calcium. 2020;85:102111. Epub 2019/12/10. doi: 10.1016/j.ceca.2019.102111 ; PubMed Central PMCID: PMC7050466. 31812825 PMC7050466

[66] Hodgin KS, Jones CL, Younger JW. Fatigue and Pain Severity in Gulf War Illness Is Associated With Changes in Inflammatory Cytokines and Positive Acute Phase Proteins. J Occup Environ Med. 2022;64(11):905–11. Epub 20220728. doi: 10.1097/JOM.0000000000002625 . 35902364

[67] Sasso EM, Muraki K, Eaton-Fitch N, Smith P, Lesslar OL, Deed G, et al. Transient receptor potential melastatin 3 dysfunction in post COVID-19 condition and myalgic encephalomyelitis/chronic fatigue syndrome patients. Mol Med. 2022;28(1):98. Epub 2022/08/20. doi: 10.1186/s10020-022-00528-y ; PubMed Central PMCID: PMC9388968. 35986236 PMC9388968

[68] Kaneko Y, Szallasi A. Transient receptor potential (TRP) channels: a clinical perspective. Br J Pharmacol. 2014;171(10):2474–507. Epub 2013/10/10. doi: 10.1111/bph.12414 ; PubMed Central PMCID: PMC4008995. 24102319 PMC4008995

[69] Zheng J. Molecular mechanism of TRP channels. Compr Physiol. 2013;3(1):221–42. Epub 2013/05/31. doi: 10.1002/cphy.c120001 ; PubMed Central PMCID: PMC3775668. 23720286 PMC3775668

[70] Khalil M, Alliger K, Weidinger C, Yerinde C, Wirtz S, Becker C, et al. Functional Role of Transient Receptor Potential Channels in Immune Cells and Epithelia. Front Immunol. 2018;9:174. Epub 2018/02/23. doi: 10.3389/fimmu.2018.00174 ; PubMed Central PMCID: PMC5808302. 29467763 PMC5808302

[71] Liu C, Montell C. Forcing open TRP channels: Mechanical gating as a unifying activation mechanism. Biochem Biophys Res Commun. 2015;460(1):22–5. Epub 2015/05/23. doi: 10.1016/j.bbrc.2015.02.067 ; PubMed Central PMCID: PMC4441759. 25998730 PMC4441759

[72] O’Boyle S, Nacul L, Nacul FE, Mudie K, Kingdon CC, Cliff JM, et al. A Natural History of Disease Framework for Improving the Prevention, Management, and Research on Post-viral Fatigue Syndrome and Other Forms of Myalgic Encephalomyelitis/Chronic Fatigue Syndrome. Front Med (Lausanne). 2021;8:688159. Epub 2022/02/15. doi: 10.3389/fmed.2021.688159 ; PubMed Central PMCID: PMC8835111. 35155455 PMC8835111

[73] Lee N, Chen J, Sun L, Wu S, Gray KR, Rich A, et al. Expression and characterization of human transient receptor potential melastatin 3 (hTRPM3). J Biol Chem. 2003;278(23):20890–7. Epub 2003/04/04. doi: 10.1074/jbc.M211232200 . 12672827

[74] Moran MM. TRP Channels as Potential Drug Targets. Annu Rev Pharmacol Toxicol. 2018;58:309–30. Epub 2017/09/26. doi: 10.1146/annurev-pharmtox-010617-052832 . 28945977

[75] Fonfria E, Murdock PR, Cusdin FS, Benham CD, Kelsell RE, McNulty S. Tissue distribution profiles of the human TRPM cation channel family. J Recept Signal Transduct Res. 2006;26(3):159–78. Epub 2006/06/17. doi: 10.1080/10799890600637506 . 16777713

[76] Thiel G, Rubil S, Lesch A, Guethlein LA, Rossler OG. Transient receptor potential TRPM3 channels: Pharmacology, signaling, and biological functions. Pharmacol Res. 2017;124:92–9. Epub 2017/07/20. doi: 10.1016/j.phrs.2017.07.014 . 28720517

[77] Wang W, Liu P, Zhang Y, Yan L, Zhu MX, Wang J, et al. Expression and functions of transient receptor potential channels in liver diseases. Acta Pharm Sin B. 2023;13(2):445–59. Epub 20220915. doi: 10.1016/j.apsb.2022.09.005 ; PubMed Central PMCID: PMC9978971. 36873177 PMC9978971

[78] Ding Q, Fang S, Chen X, Wang Y, Li J, Tian F, et al. TRPA1 channel mediates organophosphate-induced delayed neuropathy. Cell Discov. 2017;3:17024. Epub 20170801. doi: 10.1038/celldisc.2017.24 ; PubMed Central PMCID: PMC5537602. 28894590 PMC5537602

[79] Yates PL, Patil A, Sun X, Niceforo A, Gill R, Callahan P, et al. A cellular approach to understanding and treating Gulf War Illness. Cell Mol Life Sci. 2021;78(21–22):6941–61. Epub 20210927. doi: 10.1007/s00018-021-03942-3 ; PubMed Central PMCID: PMC9669894. 34580742 PMC9669894

[80] Institute of Medicine. Chronic Multisymptom Illness in Gulf War Veterans: Case Definitions Reexamined. Washington (DC) 2014. 25590117

