# Supplementary figures and images for: Novel characterization of endogenous transient receptor potential melastatin 3 ion channels from Gulf War Illness participants
Source: PLoS One. 2024 Jun 25;19(6):e0305704. doi: 10.1371/journal.pone.0305704 (PMC11198784; doi:10.1371/journal.pone.0305704)

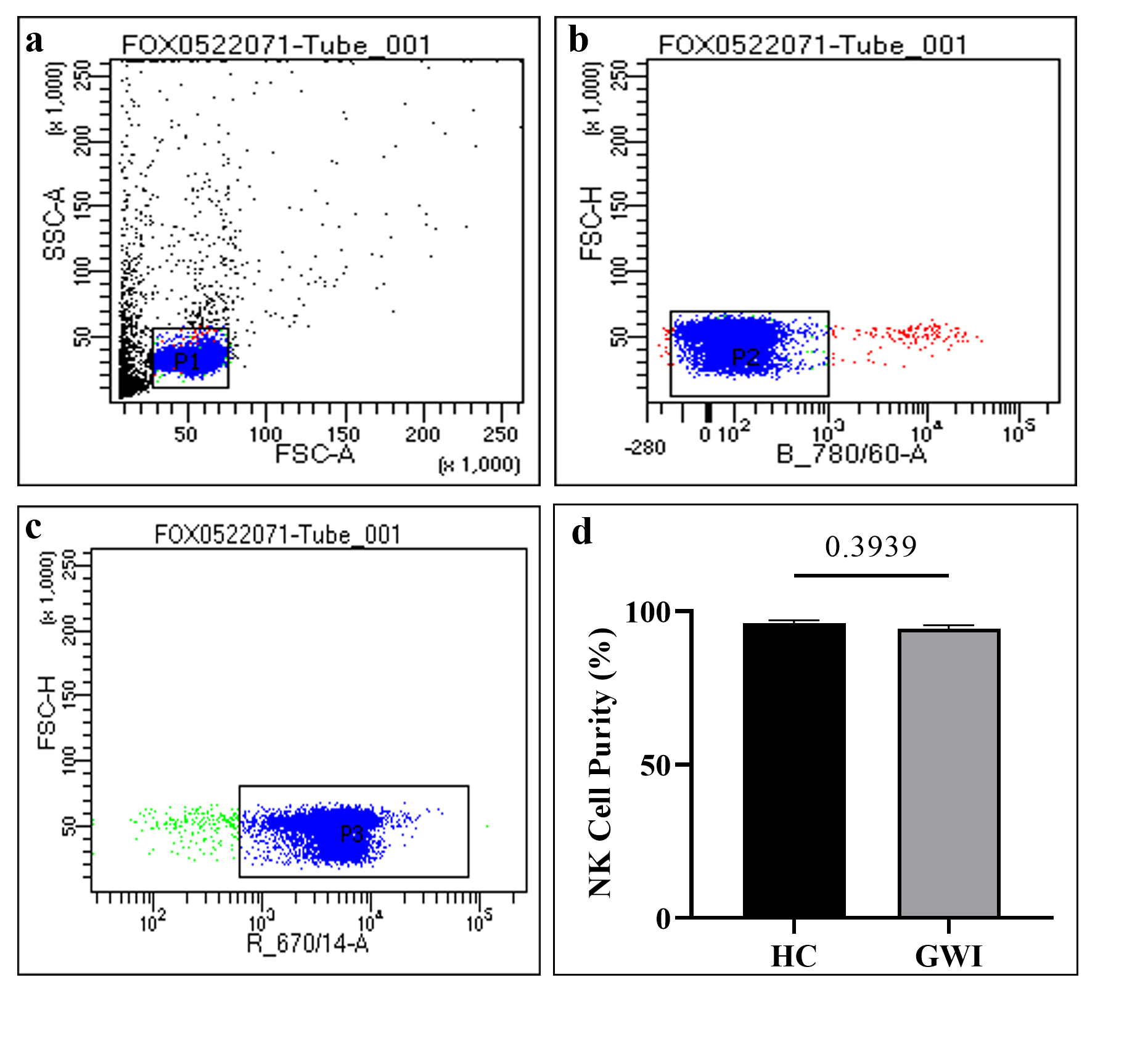

Supplement: S1 Fig — NK cell purity was acquired at 10,000 events using the BD LSRFortessaTM X-20. NK cells, defined as CD3-CD56+ lymphocytes were 96.08% ± 0.953 for HC and 94.33% ± 1.144 for participants with GWI. (a) lymphocytes were gated based on Side Scatter (SSC) and versus Forward Scatter (FSC). (b) CD3- cell population was gated from selected lymphocyte population. Gating was identified through isotype controls. (c) NK cell purity was based on CD56+ population from the CD3- population. (d) Bar graphs illustrating percentage of NK cell population. Data presented as mean ± SEM and determined by Mann-Whitney U test. Abbreviation: GWI, Gulf War Illness; HC, healthy controls; NK, natural killer. (TIF) [file pone.0305704.s001.tif]

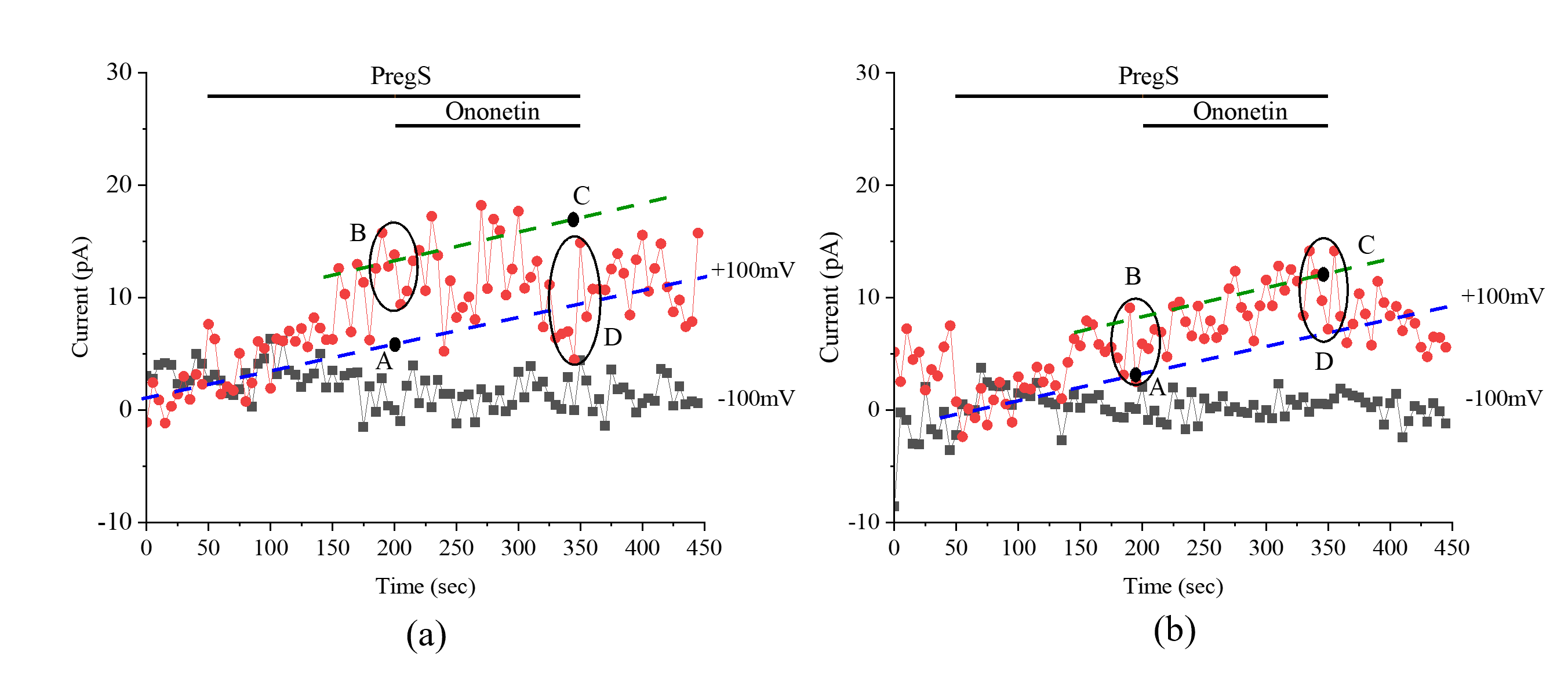

Supplement: S2 Fig — Two representative time-series of current amplitude at +100 mV and −100 mV showing the effect of PregS and Ononetin in the presence of PregS. Baseline = blue dash-lines; PregS baseline = green dash-lines. (A) Baseline; (B) PregS points; (C) PregS baseline; (D) Ononetin points. PregS amplitude = (B)–(A) and Ononetin amplitude = (C)–(D). PregS was effective when presented with an increase at +100 mV current and Ononetin was effective when there is a decrease at +100 mV current. No difference means drugs were not effective to stimulate agonist or antagonist effect on TRPM3 ion channels. On (a), PregS and Ononetin in the presence of PregS were effective, however, on (b) only PregS was effective. (TIF) [file pone.0305704.s002.tif]
